# Duration of Prion Disease is Longer in Japan Than in Other Countries

**DOI:** 10.2188/jea.JE20100085

**Published:** 2011-07-05

**Authors:** Kiwamu Nagoshi, Atsuko Sadakane, Yosikazu Nakamura, Masahito Yamada, Hidehiro Mizusawa

**Affiliations:** 1 Department of Public Health, Jichi Medical University, Tochigi, Japan; 2 Department of Neurology & Neurobiology of Aging, Kanazawa University Graduate School of Medical Science, Kanazawa, Japan; 3 Department of Neurology and Neurological Science, Tokyo Medical and Dental University, Graduate School of Medical and Dental Sciences, Tokyo, Japan

**Keywords:** prion disease, Creutzfeldt-Jakob Syndrome, epidemiology, disease duration, Japan

## Abstract

**Background:**

Prion diseases are untreatable, progressive, and fatal brain disorders that occur worldwide, and the annual incidence rate is approximately 1 case per 1 million people. The duration of these diseases in Japan is unclear.

**Methods:**

Based on data from 1 April 1999 through 4 September 2008 provided by the Japanese Creutzfeldt-Jakob disease (CJD) surveillance program, we analyzed disease duration and its relationship with clinical features. Duration was assumed to be the time from disease onset to death.

**Results:**

Evaluation by the surveillance committee indicated that during the observed period 1128 individuals received a diagnosis of prion disease and were registered in the surveillance program. Mean disease duration in the 855 patients who died was 17.4 months. Overall, 46.0% of patients died within 1 year and 77.2% died in less than 2 years. Among those with sporadic Creutzfeldt-Jakob disease, which represented 77.0% of cases, mean disease duration was 15.7 months, while that of patients surveyed by the European Creutzfeldt Jakob Disease Surveillance Network (EUROCJD) was only 5 months.

**Conclusions:**

Disease duration among Japanese with prion diseases was much longer than that of patients in Western countries conducting surveillance of prion diseases. This finding suggests that the characteristics of the system for providing life-sustaining treatment for patients with fatal, progressive diseases in Japan are related to the longer duration of these illnesses.

## INTRODUCTION

The human prion diseases (human transmissible spongiform encephalopathies) include several disease entities. Sporadic Creutzfeldt-Jakob disease (sCJD) is the most common condition and does not involve mutation of the prion protein gene (PRNP). Genetic prion diseases such as familial Creutzfeldt-Jakob disease (fCJD), Gerstmann-Sträussler-Sheinker syndrome (GSS), and fatal familial insomnia (FFI) are caused by mutation of PRNP.^1^^–^^3^ Acquired Creutzfeldt-Jakob disease is caused by abnormal prion protein, which is transmitted by cadaveric dura mater grafts (dCJD), cadaveric corneas, growth hormone prepared from human pituitary glands, and beef affected by bovine spongiform encephalopathy (variant Creutzfeldt-Jakob disease or vCJD).^1^^–^^4^ Prion diseases are progressive, untreatable, and fatal brain disorders. The gradual accumulation of abnormal prion protein causes gliosis and characteristic histologic vacuolar (spongiform) changes that result in dementia and other neurological deficits.^1^^–^^3^ Prion diseases occur throughout the world, with an annual incidence rate of approximately 1 case per 1 million people.^1^^–^^3^ In Western countries, affected patients die within 1 to 2 years.^1^^–^^3^

In Japan, rare diseases of unknown etiology or treatment, such as prion diseases, amyotrophic lateral sclerosis (ALS), systemic lupus erythematosus (SLE), and ulcerative colitis (UC), are classified as intractable diseases (excluding cancer and cardiovascular disease).^5^^–^^8^ The national program for intractable diseases was launched in 1972. Pursuant to this program, a research project (Specific Intractable Disease Research Program on Health and Labour Science Research Grants) and a registration system for medical fee subsidies (Specific Intractable Diseases Treatment Program) were developed. Prion diseases were added to the Specific Intractable Diseases Treatment Program in 1997.^8^

Surveillance systems for prion diseases were established in many Western countries during the 1990s.^10^^–^^16^ Collaborative surveillance has been conducted since 1993 by a syndicate (European Creutzfeldt Jakob Disease Surveillance Network: EUROCJD) of a number of countries (Australia, Austria, Canada, France, Germany, Italy, the Netherlands, Slovakia, Spain, Switzerland, and the United Kingdom).^17^^,^^18^ In addition, the NEUROCJD (The Extended European Collaborative Study Group of CJD) project (comprising Belgium, Denmark, Finland, Greece, Iceland, Ireland, Israel, Norway, and Portugal) was started in 1998 after the European Union Council recommended that epidemiologic surveillance of CJD should be extended to all member states.^17^

To elucidate trends in outbreaks of CJD (prion disease) in Japan, an ad hoc nationwide epidemiologic survey was conducted in 1996, when the relationship between vCJD and BSE was first reported by the United Kingdom.^4^ This survey revealed that there had been no cases of vCJD in Japan, while 43 cases of dCJD were detected.^19^ Since then, the Japanese government has administered a continuous surveillance system for CJD through the Specific Intractable Disease Research Program.^9^

The abovementioned epidemiologic characteristics of prion diseases were recently confirmed by surveillance programs in many Western countries.^10^^–^^19^ In addition, it was discovered that the duration of these diseases in Japan is longer than durations indicated in previous studies. In the present study, using data provided by the Japanese CJD surveillance program, we investigated disease duration and its relationship with patient clinical features, namely, age at onset, sex, disease subtype, primary symptoms, periodic synchronous discharge (PSD) on electroencephalograms (EEG), and PRNP codon 129 polymorphism.

## METHODS

All suspected cases of prion diseases in each prefecture are reported to the Japanese CJD Surveillance Committee, which is composed of neurologists, psychiatrists, neuropathologists, and epidemiologists. Information on patients was obtained through: (1) surveillance of infectious diseases, as authorized by the Law Concerning the Prevention of Infectious Diseases and Medical Care for Patients Suffering from Infectious Diseases, (2) application to the Specific Intractable Diseases Treatment Program, which is a further investigation of patients with intractable diseases (including prion diseases), or (3) a request from a physician-in-charge for genetic or cerebrospinal fluid (CSF) analyses by members of the surveillance committee. Each case was prospectively assessed by using a surveillance protocol that assembled information about the individual’s life history, including previous medical history, ie, any history of surgical treatment or blood transfusions, clinical history, laboratory data, and results of molecular genetic and pathologic examinations. The clinical characteristics of patients, which mainly included demographic data, clinical manifestations, presenting symptoms, and clinical examinations (EEG, CSF, and biochemical data), were also collected.

The committee confirmed or rejected the diagnosis, classified each confirmed case as sCJD, fCJD, dCJD, vCJD, GSS, or FFI, and registered it as definite, probable, or possible. Classification was based on diagnostic information (such as medical history, clinical features, EEG findings, disease duration, and neuropathologic findings). Neuropathologic confirmation, in particular, was necessary to register a diagnosis as definite. sCJD was diagnosed in accordance with the revised classical criteria established by Masters et al.^20^ In addition to the criteria for sCJD, dCJD was diagnosed after confirming a history of transplantation of cadaveric dura mater graft, and genetic prion diseases were diagnosed by confirming familial history or mutation of PRNP. A diagnosis of vCJD was based on the criteria established by the World Health Organization (WHO).^21^ Survivors were tracked twice a year by the epidemiologic center established in Jichi Medical University, using confirmation by the hospitals that had reported the cases.

There were 1906 referrals between 1 April 1999 and 4 September 2008. Among them, 1128 were registered as having received a diagnosis of a prion disease (sCJD, fCJD, dCJD, vCJD, GSS, or FFI) through evaluations at regular meetings of the committee. Of these 1128 registered patients, 862 died, 250 survived, and 16 were lost to follow-up by the day of the latest follow-up survey, conducted in September 2008. Average disease duration was calculated for the 855 decedents. Data on onset and/or death were not available for 7 patients, who were thus excluded. In the present study, definite, probable, and possible cases were analyzed. Disease duration was defined as the period from disease onset to death. For all subtypes except dCJD and FFI, the highest level of diagnostic certainty was probable. We analyzed the relationship between disease duration and other clinical features (namely, illness type, clinical classification, age at onset, sex, primary symptoms, and PSD on EEG). In addition, among the 674 patients with sCJD, 378 were analyzed to determine the prion protein genotype of codon 129 (methionine homozygote, MM; methionine/valine, MV; valine homozygote, VV).

Kaplan–Meier survival analysis was conducted for 1108 patients with data on survival status (deceased, survived, or lost to follow-up), date of onset, and date of last follow-up. Statistical analysis was conducted with SPSS version 16.0 J for Windows.

## RESULTS

Of the registered patients (*n* = 1128), the diagnoses given by the Japanese CJD Surveillance Committee were sCJD (868, 77.0%), fCJD (146, 12.9%), dCJD (70, 6.2%), GSS (37, 3.3%), FFI (3, 0.3%), vCJD (1, 0.1%), and undetermined CJD (3, 0.3%).

The distribution of fatal cases by diagnosis (excluding vCJD and undetermined CJD) is shown in Table 1. Overall, 56.7% of patients were female. As shown in Table 2, overall mean disease duration was 17.4 months. Mean disease duration among females (19.7 months) was longer than among males (14.5 months). This tendency was true for all subtypes.

**Table 1.** Classification of diseases by disease subtype and sex in fatal cases

Classification	sCJD (*n* = 674)		fCJD (*n* = 93)		dCJD (*n* = 63)		GSS (*n* = 20)		FFI (*n* = 3)	
				
	Male (*n* = 293)	Female (*n* = 381)	Male (*n* = 43)	Female (*n* = 50)	Male (*n* = 22)	Female (*n* = 41)	Male (*n* = 8)	Female (*n* = 12)	Male (*n* = 2)	Female (*n* = 1)
Definite	46 (15.7%)	44 (11.5%)	11 (25.6%)	14 (28.0%)	11 (50.0%)	17 (41.5%)	2 (25.0%)	0	2 (100%)	1 (100%)
Probable	230 (78.5%)	314 (82.4%)	32 (74.4%)	33 (66.0%)	8 (36.4%)	15 (36.6%)	6 (75.0%)	11 (91.7%)	0	0
Possible	17 (5.8%)	23 (6.0%)	0	3 (6.0%)	3 (13.6%)	9 (22.0%)	0	1 (8.3%)	0	0

Abbreviations: sCJD, sporadic Creutzfeldt-Jakob disease; fCJD, familial CJD; dCJD, CJD acquired from cadaveric dura mater grafts; GSS, Gerstmann-Sträussler-Sheinker syndrome; FFI, fatal familial insomnia.

**Table 2.** Sex and mean disease duration by disease subtype in fatal cases

	All^a^	Subtype^b^				
	
		sCJD	fCJD	dCJD	GSS	FFI
Total number (%)	855 (100)	674 (78.8)	93 (10.9)	63 (7.4)	20 (2.3)	3 (0.4)
Male (%)	370 (43.3)	293 (43.5)	43 (46.2)	22 (34.9)	8 (40.0)	2 (66.7)
Female (%)	485 (56.7)	381 (56.5)	50 (53.8)	41 (65.1)	12 (60.0)	1 (33.3)

Disease duration (months)						
Mean ± standard deviation	17.4 ± 16.7	15.7 ± 13.5	19.1 ± 18.8	19.5 ± 16.2	61.6 ± 34.4	10.6 ± 2.7
Median	13.3	12.9	13.3	14.2	49.5	12.3
Range	1–143	1–126	2–143	2–99	10–126	7–13
Mean (Male)	14.5	13.0^c^	16.6^c^	19.1^c^	44.4	9.7
Mean (Female)	19.7	17.9^c^	21.2^c^	19.7^c^	73.1	12.3

^a^Variant CJD and undetermined CJD were included.

^b^The legend for abbreviations of subtype is in Table 1.

^c^Differences in mean disease duration among 3 types of CJD (sCJD, fCJD, and dCJD) and between sexes were tested by using 2-way analysis of variance. The probabilities were 0.128 for subtype and 0.064 for sex.

Among fatal cases, 46.0% died within 1 year and 77.2% within 2 years from onset. Mean disease duration was 15.7 months for sCJD, which was the diagnosis for 77.0% of patients. The corresponding figure for GSS was 61.6 months, and 45.0% of patients with GSS lived for longer than 5 years after onset.

Among fatal cases, mean age at onset was 66.7 years (range: 15–94) and nearly 80% of patients were 60 years or older. Those with dCJD and GSS were in their 50s. In particular, more than half of patients with GSS had symptoms while in their 50s.

Regarding age at onset (–39, 40–59, 60–79, ≥80 years) for all prion diseases, earlier disease onset was associated with longer disease duration (Table 3). This trend was true for all subtypes.

**Table 3.** Age at onset and mean disease duration by disease subtype in fatal cases

Age at onset (years)	All^a^ (months) (*n* = 855)	Subtype^b^				

		sCJD (months) (*n* = 674)	fCJD (months) (*n* = 93)	dCJD (months) (*n* = 63)	GSS (months) (*n* = 20)	FFI (months) (*n* = 3)
–39	43.2 (*n* = 16)	26.8^c^ (*n* = 4)	143.4^c^ (*n* = 1)	30.5^c^ (*n* = 9)	83.4 (*n* = 2)	—
40–59	23.1 (*n* = 184)	20.7^c^ (*n* = 129)	16.5^c^ (*n* = 20)	21.9^c^ (*n* = 18)	65.3 (*n* = 12)	10.6 (*n* = 3)
60–79	15.9 (*n* = 573)	15.2^c^ (*n* = 473)	18.5^c^ (*n* = 59)	15.7^c^ (*n* = 35)	47.1 (*n* = 6)	—
80+	10.9 (*n* = 82)	9.9^c^ (*n* = 68)	15.9^c^ (*n* = 13)	11.6^c^ (*n* = 1)	—	—

^a^Variant CJD and undetermined CJD were included.

^b^The legend for abbreviations of subtype is in Table 1.

^c^Differences in mean disease duration among 3 types of CJD (sCJD, fCJD, and dCJD) and between age groups at onset were tested by using 2-way analysis of variance. The probabilities were 0.007 for subtype and 0.071 for age group.

Primary symptoms were reported in 680 cases (Table 4). Overall, 55.1% started from psychiatric symptoms (eg, dementia or sleeping difficulties), 36.5% from neurological symptoms (eg, abnormal involuntary movements or sensory disturbances, excluding ocular disturbances), 15.1% from ophthalmologic symptoms (eg, visual abnormalities), and 2.4% from other symptoms. There was no particular trend between primary symptoms and disease duration for any disease subtype.

**Table 4.** Primary symptoms and mean disease duration by disease subtype in fatal cases

Primary symptoms	All^a^ (months) (*n* = 680)	Subtype^b^			

		sCJD (months) (*n* = 535)	fCJD (months) (*n* = 77)	dCJD (months) (*n* = 50)	GSS (months) (*n* = 13)
Ophthalmologic	14.9 (*n* = 103)^c^	13.6 (*n* = 89)	28.5 (*n* = 8)	15.4 (*n* = 6)	—
Psychiatric	15.9 (*n* = 375)^c^	15.5 (*n* = 302)	17.9 (*n* = 53)	16.3 (*n* = 16)	34.3 (*n* = 1)
Neurologic	17.2 (*n* = 248)^c^	14.2 (*n* = 182)	15.4 (*n* = 16)	19.4 (*n* = 36)	59.2 (*n* = 12)
Others	18.1 (*n* = 16)^c^	58.1 (*n* = 12)	20.3 (*n* = 2)	22.7 (*n* = 1)	41.3 (*n* = 1)

^a^Variant CJD, FFI, and undetermined CJD were included.

^b^The legend for abbreviations of subtype is in Table 1.

^c^The sum of these numbers may exceed the total number of cases because more than 2 symptoms may be counted when they occur simultaneously at disease onset.

EEG examinations were performed in 840 patients, and the findings regarding the relationship between disease duration and EEG results are shown in Table 5. Of those who underwent EEG, 705 (83.9%) had PSD, which was noted in most sCJD cases. In genetic prion diseases (fCJD and GSS), the disease duration for PSD-negative patients was longer than that for PSD-positive patients.

**Table 5.** PSD^a^ on EEG^b^ and mean disease duration by disease subtype in fatal cases

PSD on EEG	All^c^ (months) (*n* = 840)	Subtype^d^				

		sCJD (months) (*n* = 665)	fCJD (months) (*n* = 90)	dCJD (months) (*n* = 62)	GSS (months) (*n* = 18)	FFI (months) (*n* = 3)
Positive	15.9 (*n* = 705)	15.5^e^ (*n* = 611)	14.5^e^ (*n* = 51)	22.9^e^ (*n* = 39)	29.5 (*n* = 2)	—
Negative	23.6 (*n* = 135)	16.1^e^ (*n* = 54)	25.6^e^ (*n* = 39)	14.1^e^ (*n* = 23)	60.5 (*n* = 16)	10.6 (*n* = 3)

^a^Periodic synchronous discharge.

^b^Electroencephalograms.

^c^Variant CJD and undetermined CJD were included.

^d^The legend for abbreviations of subtype is in Table 1.

^e^Differences in mean disease duration among 3 types of CJD (sCJD, fCJD, and dCJD) and between positive and negative PSD findings were tested by using 2-way analysis of variance. The probabilities were 0.756 for subtype and 0.864 for PSD finding.

Analyses of PRNP data from 378 patients with sCJD showed that 364 cases (96.3%) had the methionine homozygous genotype at codon 129 (MM), 11 (2.9%) were heterozygous for methionine/valine (MV), and 3 (0.8%) were homozygous for valine (VV). Disease duration was longest for the MV genotype (mean, 32.2 months vs 16.6 months for MM and 13.2 months for VV).

**Figure 1.**
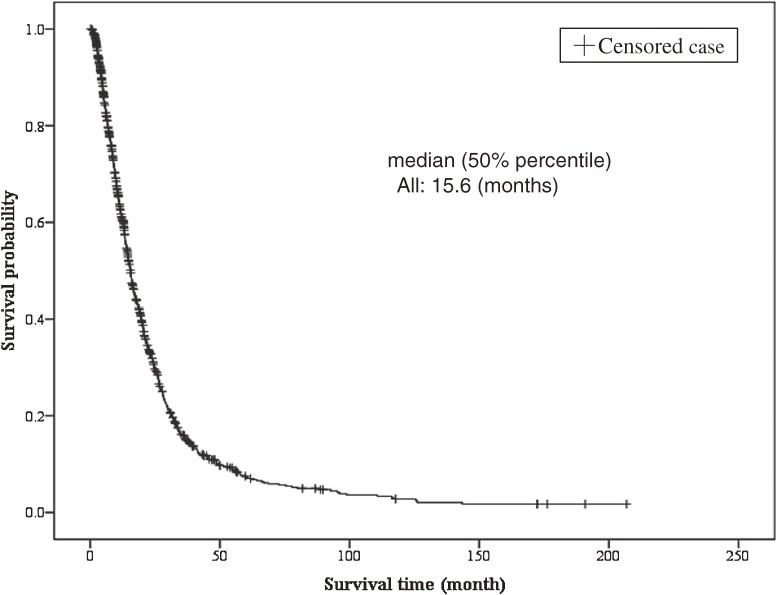
Disease duration (survival time) for all patients

The Kaplan–Meier survival curves for all patients (*n* = 1108) are shown in Figure 1. Median survival was 15.6 months. The survival rates after 60 and 120 months were 7.2% and 2.4%, respectively.

**Figure 2.**
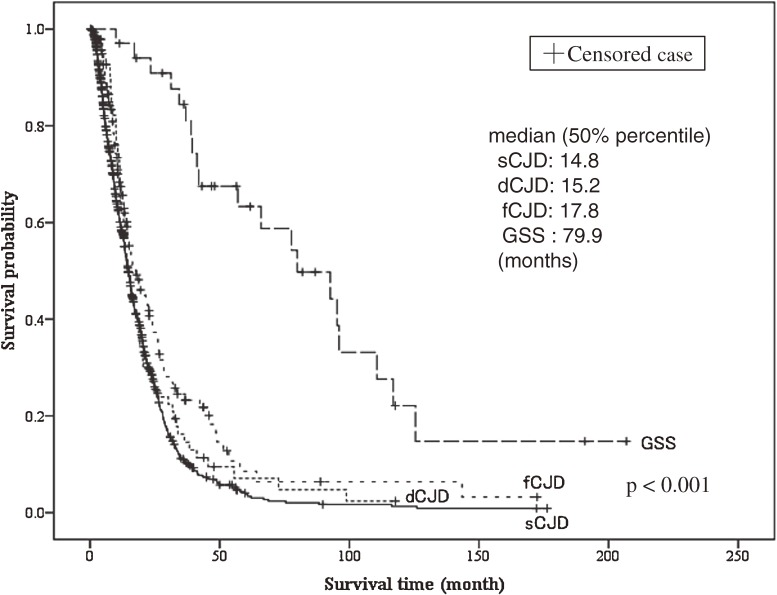
Disease duration (survival time) for patients stratified by subtype of prion disease

The survival curves for each prion disease are shown in Figure 2. Cases included in the analysis were those with sCJD (*n* = 868), dCJD (*n* = 70), fCJD (*n* = 146), and GSS (*n* = 37), and median survival was 14.8, 15.2, 17.8, and 79.9 months, respectively.

## DISCUSSION

In many countries, officials and collaborative study groups, such as EUROCJD and NEUROCJD, have developed surveillance systems for prion diseases.^10^^–^^19^^,^^22^^–^^25^ A manual for the surveillance of prion diseases was published by WHO in 2003.^26^ Because most surveillance systems, including the one in Japan, use the methods presented in the WHO manual, the reports of those surveillance systems can be compared. Therefore, an attempt was made to compare the Japanese data with other data on prion diseases.

In general, 80% to 90% of patients with prion disease have sCJD.^1^ Iatrogenic CJD (including dCJD) and fCJD each account for 5% to 10% of these cases.^1^ GSS and FFI are rare.^1^^–^^3^ This spectrum of prion diseases was also observed in Japan.^9^ The incidence of dCJD began to increase in Japan after the 1990s, indicating that the proportion of dCJD cases is greater in Japan than in Western countries.^9^^,^^19^^,^^23^

Typically, the average age at onset of sCJD and fCJD is about 60 years; for GSS and FFI, it is younger (ranging from the late 40s to the 60s).^1^ This trend was also seen in Japan.^9^

The male-to-female ratio for sCJD was reported in several countries, including Japan, and a preponderance of female cases was seen in most reports.^9^^–^^19^^,^^22^^–^^25^ In the current investigation, disease duration was longer among females, but sex differences in disease duration could not be compared because no reliable foreign data were available.

Prion diseases are invariably fatal, usually within approximately 1 year from onset. In patients with sCJD, death occurs within 1 year in 90% of cases.^1^^–^^3^ Patients usually deteriorate to a state of akinetic mutism within a few months after diagnosis.^1^^–^^3^ There is no effective therapy for preventing or treating prion diseases.^1^^–^^3^ In recent years, clinical trials have investigated both pentosan and quinine treatment; however, clinical outcomes and fundamental statistics for prion diseases were unchanged.^3^

In Japan, average disease duration is 17.4 months (range: 1–143), and 54% of patients survived for longer than 1 year. In contrast, a Swedish study found that 74.6% of patients with prion disease died within 1 year (123 patients died of prion disease between 1 January 1985 and 31 December 1996).^16^

Furthermore, average disease duration for sCJD in Japan was 15.7 months (range: 1–126), and 51.9% of patients lived longer than 1 year. In a study by EUROCJD (2451 patients who died of sCJD between 31 December 1992 and 31 December 2002), the value for the same metric was 5 months (range: 1–81), and only 14.2% of patients lived longer than 1 year.^17^^,^^18^ These findings show that the duration of prion disease in Japan was much longer than in other countries that had already implemented CJD surveillance.

The codon129 polymorphism distribution of sCJD in EUROCJD (MM type: 66.1%, MV: 17.0%, VV: 16.9%)^18^ was different from that among Japanese (especially the MM type). However, it is specious to attribute the disparity in disease duration to racial differences, as most Asians likely have the MM genotype, which is not associated with longer survival as compared with other subtypes.^27^^,^^28^

A possible reason why patients with prion diseases lived longer in Japan may be that they received much greater attention. In Western countries, due to financial and ethical concerns, intensive life-sustaining treatments like tube feeding and ventilator therapy are not commonly provided to patients with progressive, fatal neurologic condition, such as prion disease.^29^ In Japan, however, the well-organized health care system (ie, universal health insurance and free access to care) gives patients access to intensive medical treatments that prolong their lives.^30^^–^^32^ In addition, the ethical and social environment in Japan allows patients with end-stage neurologic disease to receive intensive life-sustaining treatments.^29^^,^^33^^,^^34^ Even after deterioration to akinetic mutism, many patients continue to receive life-sustaining treatment because Japanese physicians tend to have very negative attitudes toward withdrawal of life-sustaining treatment, as compared with withholding it.^35^^,^^36^

In addition to the robust public medical insurance system in Japan, under the Specific Intractable Diseases Treatment Program, the financial burden on patients and their families is reduced.^5^^–^^8^ In particular, medical treatment is free for patients with certain diseases (including prion diseases) and those in grave stages of other diseases.^8^ In this situation, many patients with a fatal neurologic disease (including prion diseases) have chosen respirator treatment under strict medical supervision.

There are several limitations in the current study. First, the new case definition of prion diseases, published in 2003,^26^^,^^37^ has not been completely incorporated into Japanese surveillance programs. In addition, the much longer disease duration in Japanese patients could cause yet another problem, especially in the diagnosis of sCJD. A new case definition of probable sCJD was introduced to the Japanese surveillance program in 2009, while a definition of possible sCJD was not. According to the new diagnostic criteria, the disease duration of possible sCJD should be shorter than 2 years. The average disease duration of possible sCJD, which constitutes 6.0% of CJD cases in Japan, was longer than 20 months, so many cases may potentially be excluded from the new diagnostic criteria. In the future, the entire Japanese CJD surveillance system will adopt the new case definition, which will result in fewer diagnoses of possible sCJD in Japan. However, the number of such cases will not be large enough to affect trends in the Japanese surveillance data.

Second, far fewer definite cases than probable cases were collected in the Japanese study, probably due to the low autopsy rate, which was only 2.7% according to Japanese Vital Statistics for 2008.^38^ However, the skill of physicians who diagnose prion disease is thought to be sufficient in Japan because information on diagnosis is disseminated via the nationwide surveillance system^6^^,^^8^^,^^9^ and because neuroimaging procedures that are required for diagnosis, such as MRI and CT, are frequently ordered.^31^ To analyze the relationship between long-term survival and the accuracy of diagnosis, the frequency of autopsies must be increased. In addition, comprehensive analysis of the details of treatment and duration of akinetic mutism is necessary if data for research is to become sufficient.

To investigate the status of prion diseases in Japan, it is necessary to use data from patients registered in the Specific Intractable Diseases Treatment Program, as well as the Japanese CJD surveillance, because most patients are enrolled in these programs.^5^^–^^8^ To receive the benefits of these programs, recipients are required to report their status annually.^8^^,^^9^^,^^39^ The data from these programs are based on information contained in the grant application form, which may not include precise clinical records. In addition, the registration system may have overlooked data from patients who died after a brief interval. However, it is useful to examine summaries of patients with intractable diseases in Japan. The mean registration period of recipients with prion diseases in 2003 (*n* = 122) was 1.5 years.^39^ This is the same as the average disease duration for fatal cases collected by Japanese CJD surveillance; therefore it is possible that disease duration in Japanese patients with prion diseases may be further extended.

In conclusion, data provided by the Japanese CJD surveillance program indicate that disease duration in patients with prion diseases is much longer in Japan than in Western countries. This suggests that the Japanese practice of providing life-sustaining treatment for patients with fatal, progressive diseases prolongs disease duration.
